# FUNGI: FUsioN Gene Integration toolset

**DOI:** 10.1093/bioinformatics/btab206

**Published:** 2021-03-27

**Authors:** Alejandra Cervera, Heidi Rausio, Tiia Kähkönen, Noora Andersson, Gabriele Partel, Ville Rantanen, Giulia Paciello, Elisa Ficarra, Johanna Hynninen, Sakari Hietanen, Olli Carpén, Rainer Lehtonen, Sampsa Hautaniemi, Kaisa Huhtinen

**Affiliations:** Research Program in Systems Oncology, Research Programs Unit, Faculty of Medicine, University of Helsinki, 00014 Helsinki, Finland; Laboratorio de Diabetes, Facultad de Química, Universidad Nacional Autónoma de México, Campus Yucatán, 97302 Mérida, México; Cancer Research Unit, Institute of Biomedicine and FICAN West Cancer Centre, University of Turku, 20014 Turku, Finland; Cancer Research Unit, Institute of Biomedicine and FICAN West Cancer Centre, University of Turku, 20014 Turku, Finland; Department of Pathology, University of Helsinki and HUS-Diagnostics, Helsinki University Hospital, 00014 Helsinki, Finland; Research Program in Systems Oncology, Research Programs Unit, Faculty of Medicine, University of Helsinki, 00014 Helsinki, Finland; Research Program in Systems Oncology, Research Programs Unit, Faculty of Medicine, University of Helsinki, 00014 Helsinki, Finland; Department of Computer Engineering, University of Modena and Reggio Emilia, 41121 Modena, Italy; Department of Engineering “Enzo Ferrari”, University of Modena and Reggio Emilia (UNIMORE), 42121 Reggio Emilia, Italy; Department of Obstetrics and Gynecology, University of Turku and Turku University Hospital, 20521 Turku, Finland; Department of Obstetrics and Gynecology, University of Turku and Turku University Hospital, 20521 Turku, Finland; Research Program in Systems Oncology, Research Programs Unit, Faculty of Medicine, University of Helsinki, 00014 Helsinki, Finland; Cancer Research Unit, Institute of Biomedicine and FICAN West Cancer Centre, University of Turku, 20014 Turku, Finland; Department of Pathology, University of Helsinki and HUS-Diagnostics, Helsinki University Hospital, 00014 Helsinki, Finland; Research Program in Systems Oncology, Research Programs Unit, Faculty of Medicine, University of Helsinki, 00014 Helsinki, Finland; Research Program in Systems Oncology, Research Programs Unit, Faculty of Medicine, University of Helsinki, 00014 Helsinki, Finland; Research Program in Systems Oncology, Research Programs Unit, Faculty of Medicine, University of Helsinki, 00014 Helsinki, Finland; Cancer Research Unit, Institute of Biomedicine and FICAN West Cancer Centre, University of Turku, 20014 Turku, Finland

## Abstract

**Motivation:**

Fusion genes are both useful cancer biomarkers and important drug targets. Finding relevant fusion genes is challenging due to genomic instability resulting in a high number of passenger events. To reveal and prioritize relevant gene fusion events we have developed FUsionN Gene Identification toolset (FUNGI) that uses an ensemble of fusion detection algorithms with prioritization and visualization modules.

**Results:**

We applied FUNGI to an ovarian cancer dataset of 107 tumor samples from 36 patients. Ten out of 11 detected and prioritized fusion genes were validated. Many of detected fusion genes affect the PI3K-AKT pathway with potential role in treatment resistance.

**Availabilityand implementation:**

FUNGI and its documentation are available at https://bitbucket.org/alejandra_cervera/fungi as standalone or from Anduril at https://www.anduril.org.

**Supplementary information:**

Supplementary data are available at *Bioinformatics* online.

## 1 Introduction

Chromosomal aberrations, such as translocations, deletions and chromosomal inversions, are common in all cancers. An important consequence of such aberrations is the fusing of two normally unrelated gene DNA segments, called fusion genes. As fusion genes are rarely present in non-malignant cells, they are considered as ideal targets for therapeutics and diagnostics purposes (Mertens et al., 2015). Fusion gene discovery has exploded due to next-generation sequencing with over 10 000 fusions currently identified. Although most of these fusions are not recurrent and are most likely passenger events, for example, fusions in ALK, ROS1, or PDGFB, or RET, NTRK1/2/3, FGFR1/2/3 and BRAF/CRAF either have targeted therapy available or are currently being tested (Schram et al., 2017).

To facilitate identification of recurrent fusion genes and fusion genes with already known therapies, we have developed FUsionN Gene Identification toolset (FUNGI). While several RNA-seq fusion calling methods have been developed (Haas et al., 2019), no gold standard for fusion gene detection exists and it has been suggested that fusion gene calling would benefit from an ensemble of methods (Gao *et al.*, 2018). FUNGI uses database annotation from FusionCatcher (Nicorici et al., 2014) and expands its application to the combined list of fusions and complements these database annotations by matching fusions to several Ensembl’s tables (paralog, homolog and GO terms) (Cunningham et al., 2019). FUNGI also provides scoring of oncogenic potential of fusions by both Pegasus (Abate et al., 2014) and Oncofuse (Shugay et al., 2013). For visualization, we have implemented a custom-made script for recreating the exact fusion and mapping the reads to it, as well as support for TrinityFusion (FusionInspector) (Haas *et al.*, 2019) for de novo assembly of fusions and remapping.

Similarly to FUNGI, FusionHub (Panigrahi et al., 2018) provides fusion database annotation, scoring with Oncofuse and visualization. The main difference with FUNGI is that FusionHub is an online resource for annotation that does not provide fusion calling, and that additionally it provides siRNA prediction based on the fusions. FUNGI is similar to nextflow based rnafusion (https://github.com/nf-core/rnafusion) in the capacity to run several fusion calling algorithms, integrating them and generating a report, but rnafusion lacks oncogenic scoring methods.

## 2 Implementation

**Fig. 1.**
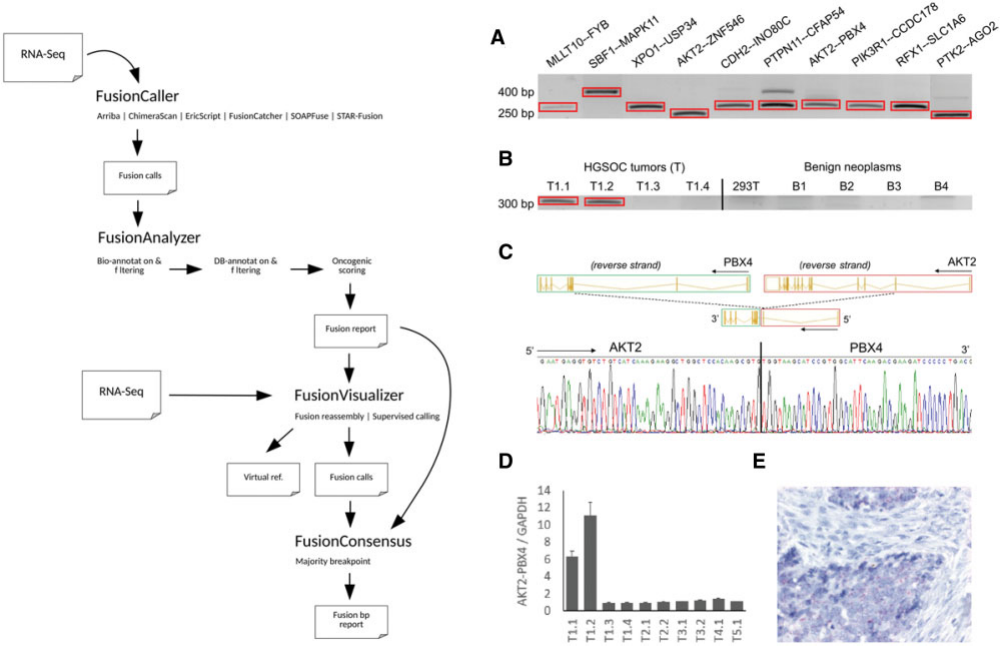
FUNGI overview and experimental validation. Left panel: FUNGI’s modules in a pipeline. Right panel: (**A**) Validation of ten gene fusions at mRNA level in the samples suggested by our computational fusion gene analysis pipeline. The fusion sites were amplified from HGSOC tissue cDNA using fusion-specific PCR and Sanger sequencing. (**B–E**) Example of further evaluation of AKT2-PBX4 fusion. (B) RT-PCR verified AKT2-PBX4 mRNA in the same two HGSOC tumor samples (T) as suggested by the RNA-seq data and in none of the benign (B) ovarian tumors sustaining pipeline specificity. (C) Sequence trace shows the specific nucleotide breakpoint of AKT2-PBX4 fusion in cDNA leading to fusion of exons 1–2 of AKT2 to exons 3–7 of PBX4. (D) RT-qPCR uncover expression of AKT2-PBX4 fusion in two out of four tumors (T1.1–T1.4) of the patient T1 as expected by the pipeline. Total of 10 individual tumors from five patients (T1–T5) were analyzed. (E) RNA in situ hybridization for AKT-PBX4 fusion reveals red fusion signal in the cancer cells while the surrounding stroma is free from the staining

FUNGI’s steps are implemented as four separate modules, which allows flexible use of the framework: modules can be run sequentially as a pipeline or as part of workflows in which users can incorporate novel fusion calling algorithms or filtering steps in between modules. The four main modules are described below, and an overview of FUNGI is shown in Figure 1.

**FusionCaller** identifies gene fusion events using RNA-Seq data. It currently supports six different fusion calling algorithms: STAR-Fusion (Haas et al., 2017), FusionCatcher, ChimeraScan (Iyer et al., 2011), EricScript (Benelli et al., 2012), SoapFuse (Jia et al., 2013) and Arriba (https://github.com/suhrig/arriba/). The output folder produced by FusionCaller can be passed on to the FUNGI’s FusionAnalyzer module directly or after method-specific filtering.

**FusionAnalyzer** filters, annotates and scores fusions coming directly from the FusionCaller module or from any other method once transformed into a standard format (example in Supplementary Data). Next, reported breakpoints are queried in Ensembl database to corroborate that the each detected fusion matches existing genes, to annotate fusion genes with Ensembl ids and to identify among them pairs of paralogs, homologs and genes with no known function to be (optionally) discarded. Remaining fusions are checked against known fusions datasets for exclusion of common artifacts or fusions previously reported in healthy individuals. After filtering, fusions are scored by both Pegasus and Oncofuse. The output from both algorithms is combined in a final report with the most relevant information from both methods. Each filtering step keeps a log of the fusions and the reasons for exclusion at every point.

**FusionVisualizer** makes a virtual reference of the fusions both with or without breakpoint information. When the coordinates of the breakpoint in each gene are provided, each chimeric transcript is reconstructed from the fusion information and then reads are mapped to it. Alternatively, FUNGI uses FusionInspector (part of STAR-Fusion) for assembling new chimeric transcripts using only the gene names and aligning reads to it. The virtual references can be loaded to a genome browser, such as IGV (Thorvaldsdóttir et al., 2013), to inspect the quality of the alignments. FusionInspector is less sensitive than normal fusion calling algorithms (Haas *et al.*, 2019), so it should be taken into account that a fusion not confirmed by supervised fusion calling is not necessarily a false positive. FusionVisualizer’s breakpoint-mode, recreating the exact fusion and mapping against it, provides useful information for manual inspection of the fusion and helps determine if it is an artifact or not. The main purpose of FusionVisualizer is to aid in rapid inspection of fusions in genome browsers and supervised fusion calling, but it is not intended to replace more sophisticated visualization tools such as Clinker (Schmidt et al., 2018) and INTEGRATE-Vis (Zhang et al., 2017).

**FusionConsensus** helps identify the best breakpoint for each fusion when there is no consensus between the tools. The breakpoint supported by the majority of methods is reported. FusionConsensus also combines fusion results from FusionAnalyzer and FusionVisualizer.

## 3 Results

We applied FUNGI to 107 RNA-seq tumor samples from 36 high-grade serous ovarian cancer (HGSOC) patients. Patient characteristics and sample processing are described in Supplementary Data. We detected 218 261 fusions, each breakpoint in each sample counted separately, that were further filtered into 228 fusion gene pairs (Supplementary Table S2). From the 228 candidate fusions, we selected 11 biologically interesting fusions for validation with cDNA Sanger sequencing (Fig. 1A, primers are listed in Supplementary Table S3). We confirmed 10 out of 11 fusions in the tumor tissues but none in benign tumors (Fig. 1B), which demonstrates that FUNGI can detect and prioritize biologically relevant fusion events when such exist in the data. RT-qPCR and RNA in situ hybridization verified the expression of AKT2-PBX4, AKT2-ZNF546 and PIK3R1-CCDC178 fusions in tumor specimens (Fig. 1D and E, Supplementary Fig. S3).

Interestingly, four of the validated fusions, i.e. AKT2-PBX4, AKT2-ZNF546, PTK2-AGO2 and PIK3R1-CCDC178, potentially affect PI3K-AKT-mTOR signaling cascade, which is constitutively hyper-activated in ∼50% of HGSOC (Mabuchi *et al*., 2015). Furthermore, for 21 fusions a gene partner belongs to PI3K-AKT-mTOR signaling cascade or regulate it (Supplementary Table S4). Three of these fusions, FGFR3-TACC3, PTK2-AGO2 (as PTK2-EIF2C2) and FTO-RBL2, have been previously reported in the Tumor Fusion Gene Data Portal (TumorFusions, https://www.tumorfusions.org/) (Hu *et al*., 2018), a web resource with fusions identified in The Cancer Genome Atlas (TCGA) dataset. For most of the remaining fusions (*n* = 19), the PI3K-pathway involved gene has been observed in a fusion with a different partner in TCGA. FGFR3-TACC3, was initially identified in glioblastoma and estimated to be present in 8% of patients. In gynecological cancers it has been reported in cervical cancer, but has not been found in breast cancer or in ovarian cancer (Costa et al., 2016). The FGFR3-TACC3 translocation predicts sensitivity to erdafitinib in urothelial cancer (Karkera *et al*., 2017).

We also used FusionVisualizer to hunt for 24 specific fusions from our result set on TCGA ovarian cancer dataset (TCGA Research Network, 2011) and we were able to reliably detect 11 fusions (FFPM≥0.1, JRC≥3; 6 additional fusions were identified below those thresholds) in several samples (Supplementary Table S5).

Taken together, we have shown the utility of FUNGI in detecting relevant fusion events from RNA-seq data. FUNGI is easy to install and has a modular design that facilitates the use of the supported fusion calling algorithms and oncogenic scoring methods, as well as integration of novel methods such as DeepPrior (Lovino et al., 2020) for oncogenic scoring. FUNGI’s Anduril (Cervera et al., 2019) implementation includes scripts for automated installation of the third-party software used. For the standalone version we have made available a Dockerfile with working testcases from which all installation steps can be consulted.

## Supplementary Material

btab206_Supplementary_Data
