# Supplementary material for: FUNGI: FUsioN Gene Integration toolset
Source: Bioinformatics. 2021 Mar 27;37(19):3353–5. doi: 10.1093/bioinformatics/btab206 (PMC8504624; doi:10.1093/bioinformatics/btab206)
Supplement: btab206_Supplementary_Data [file btab206_supplementary_data.zip › Supplementary.pdf]

Supplementary Methods

Patient and sample characteristics

A prospectively collected cohort of patients treated for high-grade serous ovarian cancer (HGSOC) was collected at Turku University Hospital between September 2010 to October 2018. All patients participating in the study gave written informed consent. The study and the use of all clinical material have been approved by The Ethics Committee of the Hospital District of Southwest Finland (ETMK) under decision number EMTK: 145/1801/2015.

We acquired 107 bulk RNA sequencing samples from 36 patients (Supplementary Table 1). Of these, 68 are primary (before chemotherapy), 32 interval (after neoadjuvant platinum-taxane chemotherapy) and 7 relapsed tumors (after being diagnosed as recurring). The samples are from primary ovarian tumors and various sites of intra-abdominal solid metastases and ascites fluid (Supplementary Figure 4).

Sanger sequencing, RT-qPCR and RNA *in situ* hybridization

Selected fusion genes were validated in HGSOC tumor specimens. Benign ovarian neoplasms were used as negative controls. Total RNA was extracted using AllPrep DNA/RNA Mini Kit (Qiagen), DNase I digested, and reverse transcribed to cDNA with oligo dT primers. The expected fusion site was amplified with fusion specific primers (Supplementary Table 3) and Sanger sequenced.

Expression of selected fusion genes was quantitated in triplicate cDNA samples using CybrGreen RT-qPCR and normalized to GAPDH. RNA *in situ* hybridization with BaseScope assay was carried out to visualize fusion gene expression according to the manufacturer's instructions (Advanced Cell Diagnostics, Newark, CA, USA). We utilized customized BaseScope probes for AKT2-PBX4 (#719661), AKT2-ZNF546 (#719671) and PIK3R1-CCDC178 (#719681) fusions, as well as BaseScope Positive Control Probe Hs-PPIB-1ZZ (#701041) and Negative Control Probe-DapB-1ZZ (#701021). In brief, formalin fixed paraffin embedded tissue sections were deparaffinated, treated with target retrieval reagents at 98°C for 15 min, and digested with protease IV for 15 min at 40°C in the hybridization oven. The slides were next hybridized with the BaseScope probes for 2 h at 40°C, followed by a serial amplification steps at 40°C in the hybridization oven or at room temperature as instructed, and finally incubated with the Fast Red substrate at room temperature to visualise the hybridization signals. The stained slides were digitalized using 3DHISTECH Pannoramic 250 FLASH II digital slide scanner at Genome Biology Unit supported by HiLIFE and the Faculty of Medicine, University of Helsinki, and Biocenter Finland.

RNA-Seq fusion calling and processing

FUNGI's (version 1.0) FusionCaller, FusionAnalyzer and FusionVisualizer were used for calling parameters used are described below.

For identifying the fusion genes we used FusionCaller with no pre-processing of the RNA-seq reads, default parameters, and genome version GRCh38 coordinates. We used 5 fusion calling algorithms from FusionCaller: Arriba (v.1.2), SoapFuse (v1.27), FusionCatcher (v1.00), EricScript (v0.5.5), ChimeraScan (v.0.4.6) and STAR-Fusion (v1.1.0). Fusions were filtered depending on the caller as follows: score > 0.5 (EricScript), Counts_of_common_mapping_reads < 30 (FusionCatcher), Overlapping_Same = true (ChimeraScan), and LargeAnchorSupport=YES_LDAS (STAR-Fusion). FusionAnalyzer was used for combining fusions in a standardized format and removing highly probable false positives (fusions between paralog/homolog genes, not matching Ensembl gene coordinates or neither gene having a known function) and fusions annotated as bodymap2, cacg, conjoining, cta, ctb, ctc, ctd, duplicates, overlapping, fragments, gtex, metazoa, mirna, mt, non_cancer_tissues, non_tumor_cells, pair_pseudo_genes, paralogs, rp11, rp, rrna, similar_reads, similar_symbols, yrna, pairs_pseudogenes, no_protein, healthy, conjoing, bodymap, 1000genomes. List of databases and accession date included below. Remaining fusions were scored with Pegasus (installation instructions in https://bitbucket.org/alejandra_cervera/fungi/ show how to update Pegasus for usage with GRCh38) and Oncofuse [v1.1.1]. Thresholds were 0.25 and 0.6 for Pegasus and Oncofuse scores respectively. Fusions detected only by EricScript were discarded as well as fusions detected in non-cancer databases but not in cancer databases. The remaining fusions were fed to our FusionVisualizer component that uses FusionInspector (included in STAR-Fusion_v2.7.0f_0328) to perform in silico validation. We removed fusions with a Junction Read Count (JRC) <3, no Large Anchor Support, and rounded FFPM<0.1.

Databases

Ensembl database version: 86; Organism: Homo sapiens; Genome version: GRCh38

Databases obtained through FusionCatcher:

- NCBI Viral Genomes version: 2017-09-15
- RefSeq NCBI database version (downloaded from UCSC database; hg38): 2017-10-29
- Gencode database version: 27
- Non-cancer tissues and cells (Babiceanu et al. Nucl. Acids Res. 2016) database version: 2017-11-09
- Cancer Gene List database version: 2017-11-09
- Oncogenes database version: 2017-11-09
- TumorFusions (Hu et al. Nucleic Acids Research) database version: Nov. 2017
- Cell lines (Klijn et al. Nature Biotechnology 2014) database version: 2017-11-09
- Prostate Tumor Patients (Robison et al. Cell 2015) database version: 2017-11-09
- Pancreatic Tumor Patients (Bailey et al. Nature 2016) database version: 2017-11-09
- ChimerDB database version: 2.0
- ChimerDB database version: 3.0
- TICdb database version: 2017-11-09

- ConjoinG database version: 2017-11-09
- CGP database version: 2017-11-09
- CACG database version: 2017-11-09
- DGD database version: 2017-11-09
- GTEx database version (thru FusionAnnotator): 2017-11-09
- TumorFusions (Hu et al. Nucleic Acids Research) database version: Nov. 2017

TCGA fusion calling

After downloading level 1 data from The Cancer Genome Atlas (TCGA) (The Cancer Genome Atlas Research Network, 2011) we searched for selected fusions on 424 samples from 417 patients using FusionVisualizer with FusionInspector (included in STAR-Fusion_v2.7.0f_0328).

FusionAnalyzer standard input format

To input fusions not called using FusionCaller with any of the 6 supported methods (FusionCatcher, ChimeraScan, STAR-Fusion, EricScript, Arriba or SoapFuse), the fusions need to be submitted in a tab separated file with the following columns:

- Left-gene name
- Right-gene name
- Left-gene ensembl-id
- Right-gene ensembl_id
- Left-gene start-position:Left-gene end-position:Left-gene strand
- Right-gene start-position:Right-gene end-position:Right-gene strand
- Left-gene chromosome:breakpoint-Right-gene chromosome:breakpoint
- Spanning reads count
- Encompassing reads count

Example of csv file:

gene1_name	gene2_name	ensembl_id1	ensembl_id2	start1:end1:strand1	start2:end2:strand2	chr1:bp1-chr2:bp2	span_cnt	split_cnt
ACTN3	CTSF	ENSG00000248746	ENSG00000174080	66546395:66563329:+	66563463:66568841:-	chr11:66563329-chr11:66563882	4	3
ADAMTS7	TCHP	ENSG00000136378	ENSG00000139437	78759203:78811431:-	109900264:109983841:+	chr15:78788231-chr12:109933637	4	3
APPBP2	SEMA3E	ENSG00000062725	ENSG00000170381	60443149:60526219:-	83363906:83649010:-	chr17:60494466-chr7:83490271	64	26
B4GALT7	ASXL2	ENSG0000027847	ENSG00000143970	177600100:177610347:+	25733753:25878516:-	chr5:177610347-chr2:25743797	5	3

If any of the six supported methods were used but not with FusionCaller then add two files (.sample and .tool) to the output folder of one of the six supported tools and there is no need to reformat the fusion calls. Both files are just have one line; .sample file has only the sample id name, and .tool has any of the following: arriba, chimerascan, ericscript, fusioncatcher, soapfuse, or starfusion.

Pipeline running time

Each different fusion calling algorithm has different running times, below we have included information on the running time with standard parameters for each method for one sample. For our largest sample (3217M, 62,937,802 read pairs) the fusion calling times for each method:

- SOAPFuse 16 hrs (4 threads)
- EricsSript 21 hrs (4 threads)
- ChimeraScan 26 hours (8 threads)
- STAR-Fusion 2 hours (4 threads)
- FusionCatcher 7 hours (4 threads)
- Arriba 5 hours (8 threads)

For the FusionAnalyzer step, the running time depends on the number of fusions to be integrated and to lesser degree the number of samples from which the fusions come from. In our case of 216,514 the running time was an estimated 17 hrs.

FusionVisualizer running FusionInspector the running time was 18 hours.

Supplementary Figures

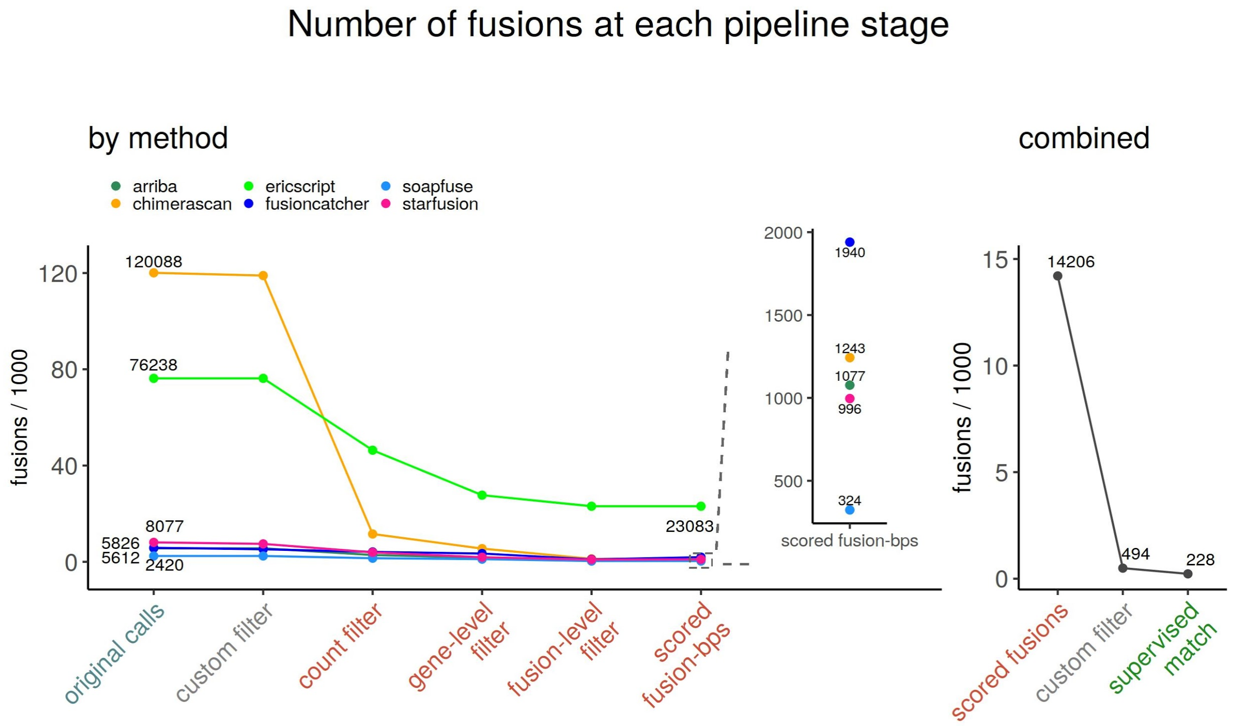

Supplementary Figure 1. Number of fusions called by each of the six methods on 107 RNA-seq samples. On the left, the number of fusions is shown independently for each method. Steps in blue relate to FusionCaller, in red FusionAnalyzer, and in green FusionVisualizer module. Custom filter refers to filtering steps in between FUNGI modules, on the left panel is a method-specific filtering, on the right, custom filter refers to filtering of fusions based on oncogenic score, unique to EricScript and annotated in healthy tissue databases while not in cancer databases (filtering steps are described in full in the text and Supplementary). For *by method* (left) plot each reported fusion is counted separately (each breakpoint sample combination is counted as one fusion), while on the *combined* plot (right) only distinct fusion gene-pairs are counted.

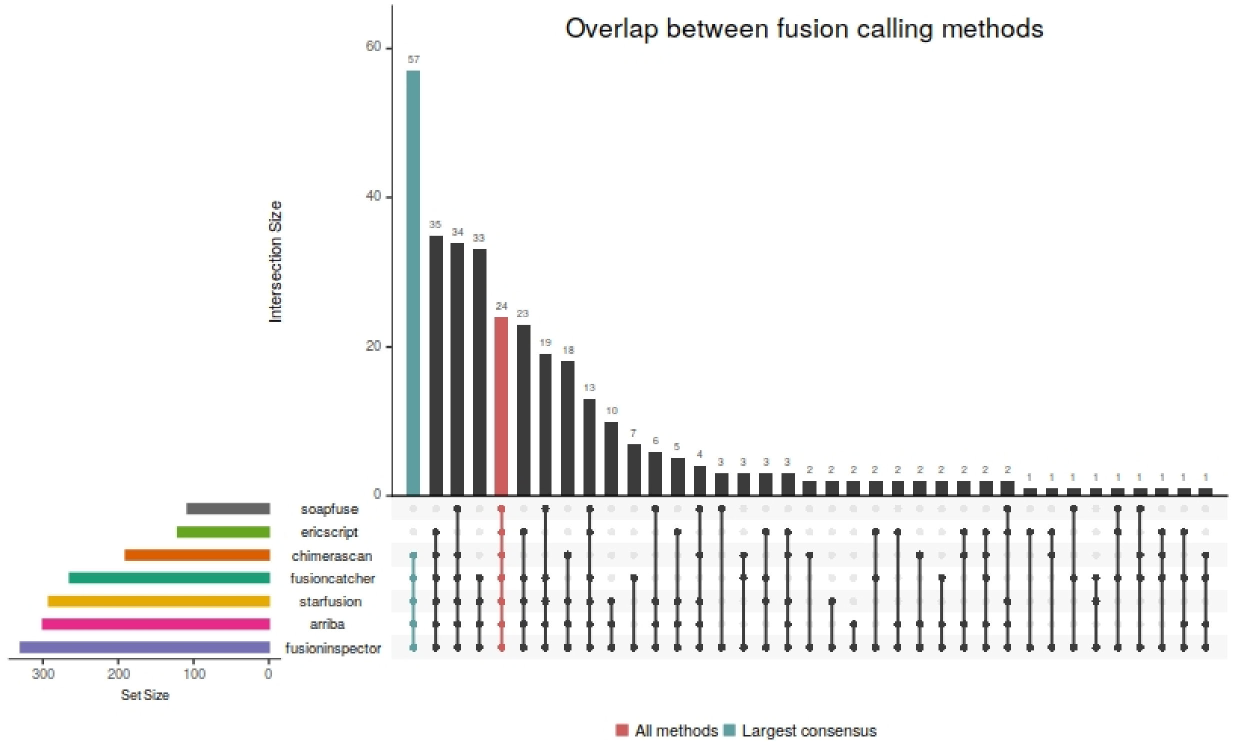

Supplementary Figure 2. Overlap of fusions detected by each method on the same sample.

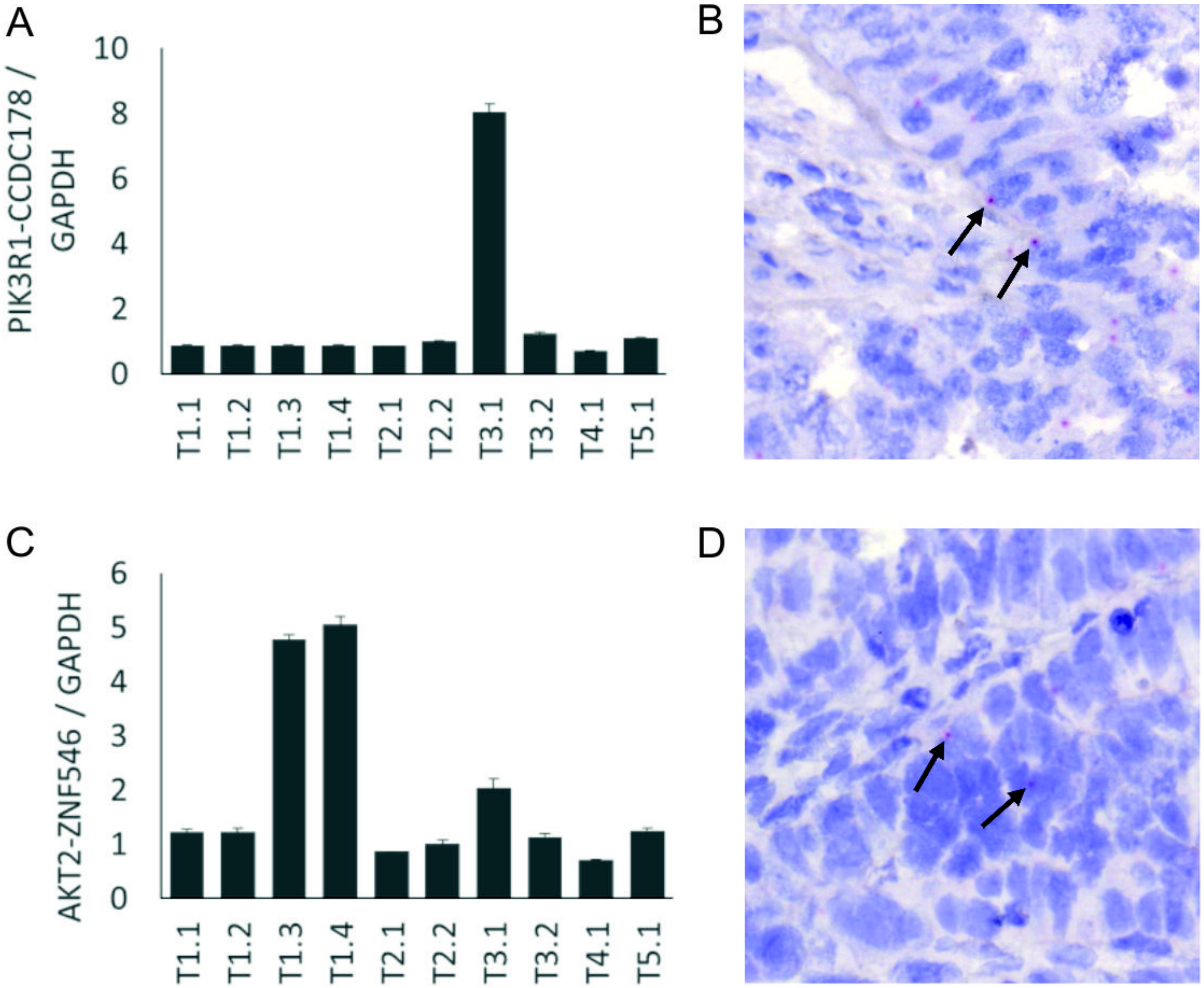

Supplementary Figure 3. Expression and localization of the PIK3R1-CCDC178 and AKT2-ZNF546 fusions at the mRNA level in patient tumors. A) The RNA-seq data suggested the presence of the PIK3R1-CCDC178 fusion in patient samples T3.1 and T3.2. Fusion expression was confirmed with RT-qPCR in T3.1. B) However, the fusion was detected in both samples by RNA in situ hybridization. C) The AKT2-ZNF546 fusion was strongly expressed in both of the suggested samples, T1.3 and T1.4. D) The fusion signal was weakly present in one of the two samples by RNA in situ hybridization. The black arrows point to tumor cells with the fusion.

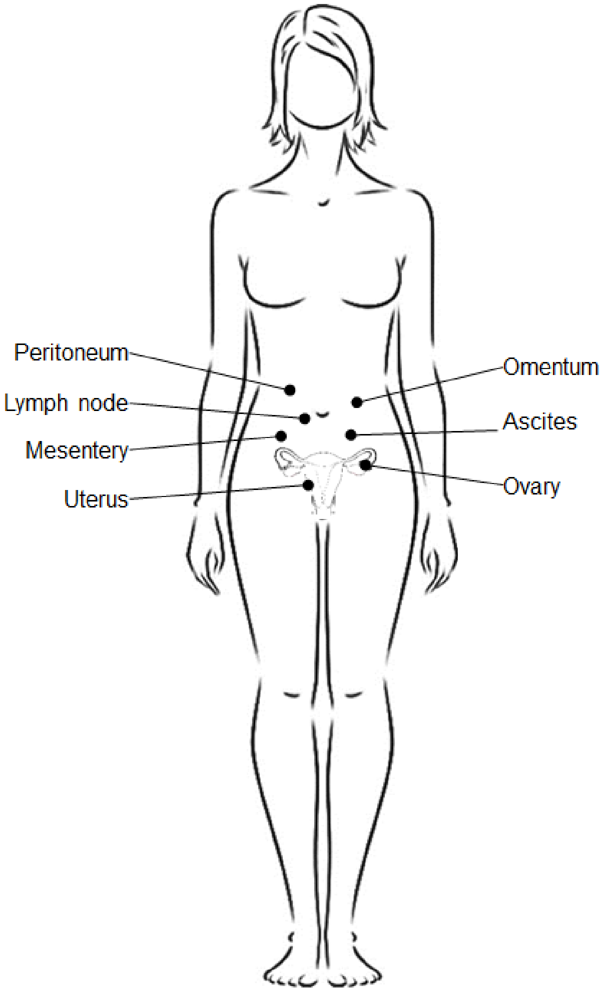

Supplementary Figure 4. Primary high-grade serous ovarian tumors and various sites of intra-abdominal solid metastases and ascites fluid were analyzed by FUNGI.

Supplementary Tables

Supplementary Table 1. Patient characteristics and sample read sizes.
Supplementary Table 2. Fusion result list from FUNGI on our sample set.
Supplementary Table 2. Primers used to validate fusions using Sanger sequencing.
Supplementary Table 4. Fusions with a gene in PI3K-AKT pathway.
Supplementary Table 5. Supervised fusion calling in TCGA.
